# Correlation between blood flow on optic nerve head and structural and functional changes in eyes with glaucoma

**DOI:** 10.1038/s41598-020-57583-w

**Published:** 2020-01-20

**Authors:** Fumi Kuroda, Takeshi Iwase, Kentaro Yamamoto, Eimei Ra, Hiroko Terasaki

**Affiliations:** Department of Ophthalmology, Nagoya University Graduate School of Medicine, Nagoya, Japan

**Keywords:** Medical imaging, Outcomes research

## Abstract

The purpose of this study was to determine the significance of the correlations between blood flow on the optic nerve head (ONH) using the mean blur rate (MBR) determined by laser speckle flowgraphy and the visual field loss determined by perimetry and the structural alterations by optical coherence tomography in eyes solely with open-angle glaucoma. There were significant differences in the circumpapillary retinal nerve fiber layer thickness (cpRNFLT), and the MBR-tissue, at the different stages of glaucoma (ANOVA, *P* < 0.001). Univariate linear regression analyses indicated that the mean deviations (MD) were significantly correlated with both the MBR-tissue (r = 0.661, *P* < 0.001) and the cpRNFLT (r = 0.279, *P* = 0.005). Logistic regression analyses showed that the MD was significantly correlated with the MBR-tissue (*P* < 0.001) and the cpRNFLT (*P* < 0.001). The MBR-tissue was found to be the factor that can best predict the MD based on the Akaike information criteria (*P* < 0.001). Stepwise multiple logistic regression analyses showed that the MBR-tissue and the cpRNFLT were both risk factors that were significantly associated with the MD (Odds ratio;1.25 and 1.07, *P* < 0.001 and *P* < 0.001, respectively). These results indicate that the MBR-tissue was as important as the structural values in diagnosing and determining the prognosis of glaucoma.

## Introduction

Glaucoma is a group of multifactorial ophthalmic diseases that is characterized by a progressive reduction of retinal ganglion cells (RGCs) leading to pathognomonic optic nerve head (ONH) damage and optic neuropathy with corresponding defects in the visual field^1^. Glaucoma can be distinguished from other progressive optic neuropathies by characteristic changes in the cupping of the ONH and by a reduction in visual function mainly away from the center of the visual field^1^.

The relationships between the structure and function in glaucomatous eyes have been examined extensively, and in more detail recently by spectral domain-optical coherence tomography (SD-OCT). There have been reports that eyes with early stage glaucoma have a relatively larger number of RGCs loss before a measurable change in the mean deviations (MD) of the visual fields can be detected^2,3^. OCT has been found to be an important method to detect pre-perimetric glaucoma (PPG) which is defined to be present when no visual field defects are detected in spite of the existence of distinguishing glaucomatous changes in the ONH and damage of the retinal nerve fiber layer (RNFL)^4,5^. At advanced stages of glaucoma, a small additional reduction in the RGC numbers can result in a significant visual field loss. SD-OCT measurements of the tissue thicknesses are not useful for determining the stage of glaucoma because of the ‘floor effect’, i.e., the RNFLT has a lower limit after which no additional thinning can occur^6–8^. This floor effect is considered to be a serious limitation for monitoring the structural changes in eyes with advanced glaucoma, and it would be better to find other ways to determine the progression of glaucoma especially in eyes with advanced glaucoma.

The results of a number of studies have suggested that abnormalities in the ocular and systemic blood flows, especially on the ONH, play important roles in the development and progression of glaucoma^9–11^. However, the actual causation of those parameters has not been published. Thus, a better understanding of the blood flow abnormalities on the ONH should greatly assist in the identification of the factors that influence the pathophysiology and progression of glaucoma.

A number of techniques have been developed to evaluate the blood flow on the ONH but technical limitations have hindered imaging the posterior regions of the eye and the ONH. Recent advancements in laser speckle flowgraphy (LSFG) have allowed this technique to obtain valuable new information on the blood flow in these regions of the eye. LSFG can measure the relative blood flow of the vessels on the ONH and choroid noninvasively and without the use of contrast agents^12^. LSFG studies have shown that the mean blur rate (MBR), a measure of the blood flow rate, is reduced in eyes with glaucoma^13^. While there is consensus that ocular blood flow is reduced in glaucomatous eyes, the exact relationships between the functional changes, i.e., visual field loss, and the structural damages and the blood flow on the ONH have not been definitively determined.

Thus, the purpose of this study was to determine the significance of the correlations between the MBR on the ONH and the visual field loss and the structural alterations of the retina. To accomplish this, we determined the MBR on the ONH by LSFG and the structural alterations by OCT and visual field defects by perimetry.

## Results

### Demographics of patients

Thirty eyes of 30 patients with PPG and 105 eyes of 105 patients with OAG were age-matched with 30 normal eyes of 30 patients. The baseline clinical characteristics of the five study groups, viz., control group, PPG group, mild OAG group, moderate OAG group, severe OAG group, and their comparative *P* values are shown in Table 1. The differences in the age, refractive error, axial length, cardiovascular variables, e.g., systolic blood pressure (SBP), diastolic blood pressure (DBP), mean arterial blood pressure (MAP), mean ocular perfusion pressure (MOPP), and heart rate (HR), trabeculotomy ratio, and systemic conditions, e.g., hypertension, were not significant among the five groups. The sex distribution (*P* = 0.002), IOP (*P* = 0.014), the MD (*P* < 0.001), circumpapillary retinal nerve fiber layer thickness (cpRNFLT) (*P* < 0.001), macular ganglion cell layer plus inner plexiform layer thickness (mGCIPLT) (*P* < 0.001), ONH MBR-tissue (*P* < 0.001), ONH-MBR-vessel (*P* < 0.001), MBR-all (*P* < 0.001), trabeculectomy ratio (*P* < 0.001), pseudophakia ratio (*P* = 0.016), intracranial disease ratio (*P* = 0.040), number of anti-thrombotic medications (*P* = 0.011), and glaucoma medications (*P* < 0.001) were significantly different among the five groups. Additionally, diabetes mellitus was approaching significance (*P* = 0.052)

**Table 1:** Clinical characteristics of the control, preperimetric glaucoma and OAG groups.

Variable	Control	Preperimetric Glaucoma	OAG			P value
			Mild	Moderate	Severe	
Number of eyes	30	30	35	29	41	—
Sex (m/f)	12/18	10/20	17/18	15/14	32/9	0.002
Age (years)	68.1 ± 11.8	66.3 ± 10.5	71.3 ± 11.0	69.1 ± 13.9	71.4 ± 8.7	0.070
BCVA (logMAR)	0.05 ± 0.08	0.05 ± 0.08	0.03 ± 0.06	0.06 ± 0.09	0.16 ± 0.22	<0.001
Refraction (diopter)	−1.25 ± 2.06	−2.26 ± 2.22	−2.07 ± 2.14	−2.21 ± 1.96	−2.38 ± 2.26	0.342
Axial Length (mm)	24.29 ± 0.92	25.11 ± 1.22	24.43 ± 0.98	24.86 ± 1.74	25.01 ± 1.55	0.610
IOP (mmHg)	15.2 ± 2.9	13.8 ± 2.4	13.7 ± 3.1	12.7 ± 4.4	12.5 ± 3.4	0.014
MD (dB)	0.49 ± 0.58	−0.49 ± 0.78	−3.20 ± 1.41	−9.10 ± 1.54	−18.97 ± 5.26	<0.001
cpRNFLT(µm)	96.32 ± 10.85	81.43 ± 7.92	73.17 ± 9.63	64.41 ± 9.32	62.66 ± 4.75	<0.001
mGCIPLT (µm)	79.83 ± 8.57	73.03 ± 9.67	67.47 ± 9.60	62.38 ± 8.63	60.70 ± 10.99	<0.001
SBP (mmHg)	131.2 ± 20.9	133.0 ± 20.0	128.1 ± 16.2	131.1 ± 18.1	131.3 ± 22.3	0.842
DBP (mmHg)	75.9 ± 12.2	78.8 ± 10.9	73.9 ± 11.6	77.5 ± 12.1	75.9 ± 13.4	0.401
MAP (mmHg)	94.4 ± 14.6	96.9 ± 13.1	91.9 ± 12.6	95.4 ± 13.7	94.4 ± 16.0	0.589
MOPP (mmHg)	47.7 ± 9.2	51.2 ± 8.6	47.6 ± 8.6	50.9 ± 8.9	50.5 ± 10.7	0.206
HR (bpm)	72.3 ± 10.8	73.3 ± 10.9	72.6 ± 8.8	70.0 ± 8.6	72.5 ± 10.9	0.350
Choroidal MBR	8.28 ± 2.24	8.71 ± 2.95	9.35 ± 2.96	9.29 ± 2.83	7.76 ± 2.24	0.048
ONH MBR- vessel (AU)	40.44 ± 7.63	38.82 ± 9.61	36.05 ± 7.56	30.85 ± 9.36	27.02 ± 7.48	<0.001
ONH MBR- tissue (AU)	10.89 ± 2.47	10.63 ± 2.10	10.19 ± 1.69	9.37 ± 1.89	6.95 ± 1.61	<0.001
ONH MBR- overall (AU)	21.01 ± 4.09	19.65 ± 4.95	17.65 ± 3.82	15.20 ± 3.88	12.23 ± 3.35	<0.001
Trabeculectomy (n/%)	0/0.0	0/0.0	4/11.4	8/27.6	30/73.1	<0.001
Trabeculotomy (n/%)	0/0.0	0/0.0	0/0.0	2/6.9	2/4.9	0.236
Pseudophakia (n/%)	8/26.7	11/36.7	17/48.6	14/48.3	27/65.9	0.016
Diabetes mellitus disease (n/%)	11/36.7	10/33.3	10/28.6	10/34.5	22/53.7	0.052
Hypertension disease (n/%)	12/40.0	12/40.0	14/40.0	10/34.5	16/39.0	0.991
Intracranial disease (n/%)	4/13.3	0/0.0	0/0.0	2/6.9	5/6.1	0.040
Antithrombotic medication (n/%)	1/3.3	7/23.3	1/2.9	2/6.9	9/22.0	0.011
glaucoma medication (n/%)	0/0.0	13/43.3	31/88.6	20/69.0	33/80.4	<0.001

OAG: open angle glaucoma, BCVA: best-corrected visual acuity, logMAR: logarithm of the minimum angle of resolution,

IOP: intraocular pressure, MD: mean deviation, cpRNFLT: circumpapillary retinal nerve fiber layer thickness,

mGCIPLT: macular ganglion cell inner plex layer thickness, SBP: systemic blood pressure, DBP: diastolic blood pressure,

MAP: mean arterial blood pressure, MOPP: mean ocular perfusion pressure, HR: heart rate, ONH: optic nerve head, MBR: mean blur rate.

AU: arbitrary units.

### Differences in structure and values of blood flow parameters among 5 groups

**Figure 1:**
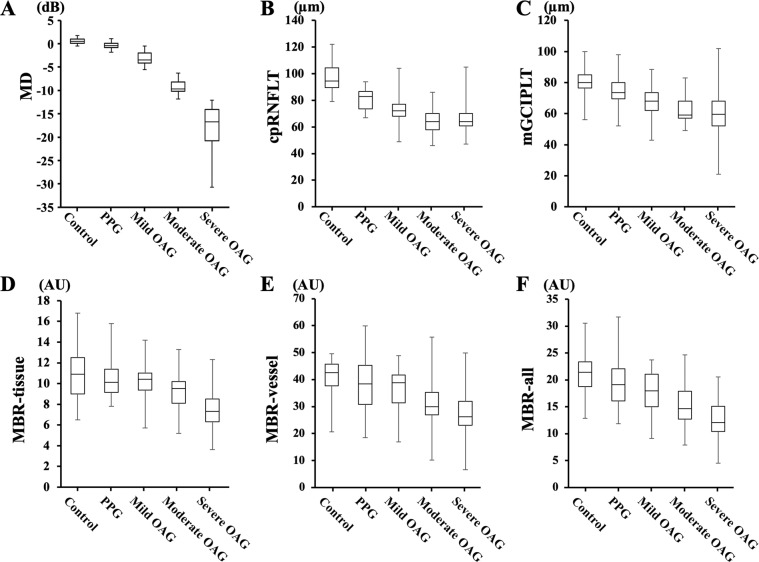
Box-and-whisker plot showing differences in the mean deviations (**A**: MD), the circumpapillary retinal nerve fiber layer thickness (**B**: cpRNFLT), the ganglion cell layer plus inner plexiform layer thickness (**C**: mGCIPLT), and the mean blur rate (MBR)-tissue (**D**), MBR-vessel (**E**), and MBR-all (**F**). The bottom and top of the box are the first and third quartiles, and the band within the box is the median. The ends of the whiskers represent the minimum and maximum of all the data.

Box-and-whisker plot of the MD, cpRNFLT, mGCIPLT, and MBRs for the five study groups are shown in Fig. 1. There were significant differences in the MD, the cpRNFLT, the mGCIPLT, and the MBR-tissue, MBR-vessel, and MBR–all among the five groups *(P* < 0.001).

### Relationships between mean deviation (MD) of visual fields and structural changes or values of blood flow parameters

Univariate linear regression analysis showed that the MD was significantly correlated with the MBR-tissue (r = 0.661, *P* < 0.001), MBR-vessel (r = 0.416, *P* < 0.001), MBR-all (r = 0.550, *P* < 0.001), cpRNFLT (r = 0.279, *P* = 0.005), and mGCIPLT (r = 0.393, *P* < 0.001). Because the MBR-tissue had the highest correlation with the MD, the MBR-tissue was used for further analyses.

Multivariate linear regression analyses showed that the MBR-tissue (β = 0.427, *P* < 0.001), cpRNFLT (β = 0.422, *P* < 0.001), and BCVA (β = −0.197, *P* = 0.006) were independent factors significantly correlated with the MD (Table 2).

**Table 2:** Multiple regression analysis of variables affecting MD.

Dependent variable	Independent variables	β	P value
MD	MBR-tissue	0.427	<0.001
	cpRNFLT	0.422	<0.001
	logMAR BCVA	−0.197	0.006
	mGCIPLT	0.128	0.164
	Age	−0.09	0.192
	OPP	0.071	0.31
	AL	−0.069	0.332
	IOP	0.009	0.902

**Figure 2:**
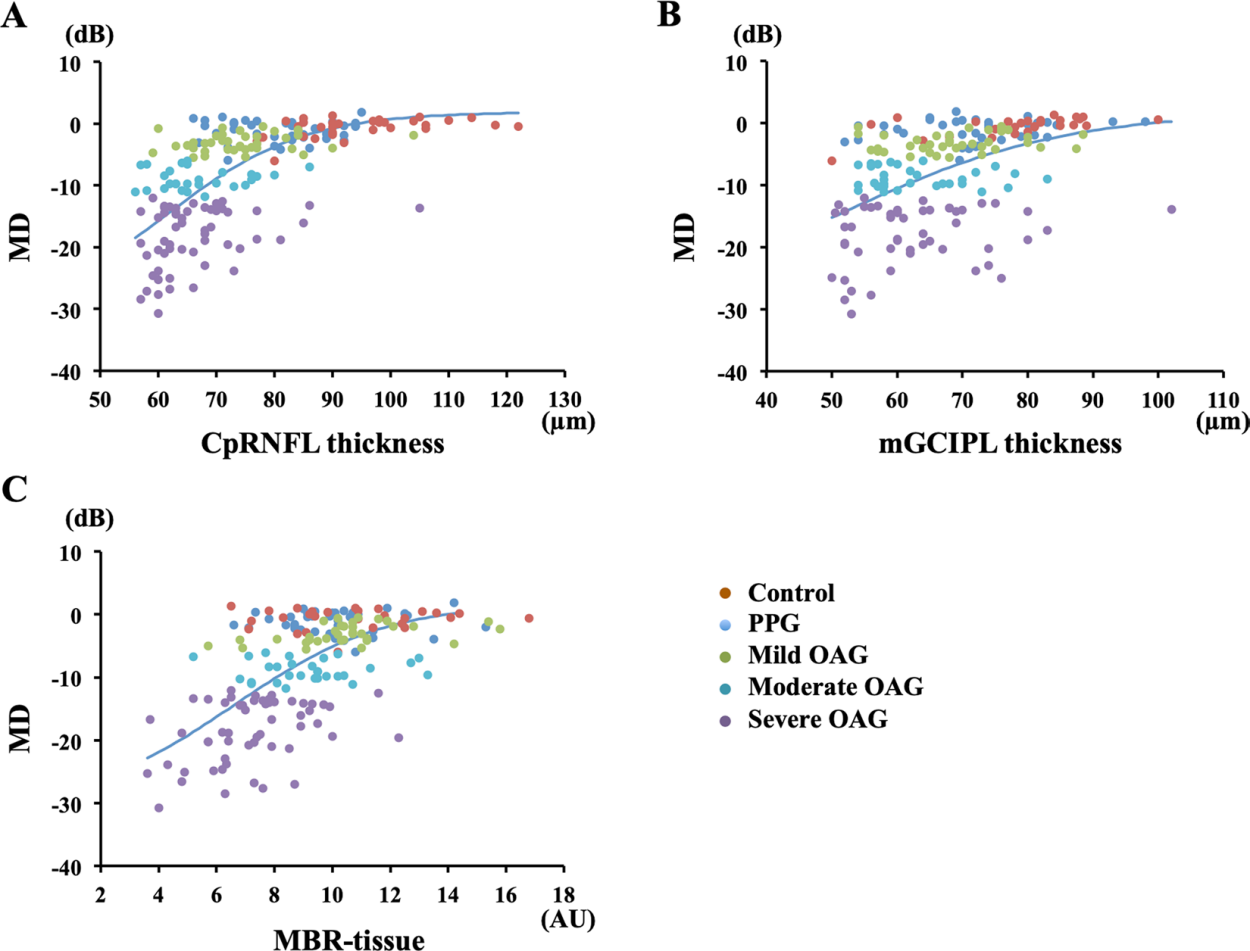
Logistic regression analysis of the relationship between the MD and the cpRNFLT, the mGCIPLT, and the MBR-tissue. There were significant correlations between the MD and the cpRNFLT (**A**), the mGCIPLT (**B**), and the MBR-tissue (**C**).

The results of the logistic regression analyses on the relationship between the MD and the cpRNFLT, mGCIPLT, and MBR-tissue are shown in Fig. 2. There were significant correlations between the MD and the MBR-tissue (*P* < 0.001), the cpRNFLT (*P* < 0.001), and the mGCIPLT (*P* = 0.002). The MBR-tissue can be best described for the MD based on the Akaike information criterion (AIC) using logistic regression analysis (AIC = 142.22, *P* < 0.001; Table 3).

**Table 3:** Logistic regression analysis of the relation between MD and other factors.

Factor	Coefficient	P-value	AIC
MBR-tissue	0.34871	<0.001	142.22
cpRNFLT	0.0819	<0.001	142.31
LogMAR	−2.8137	0.0168	171.45
mGCIPLT	0.0553	0.0019	152.17

**Figure 3:**
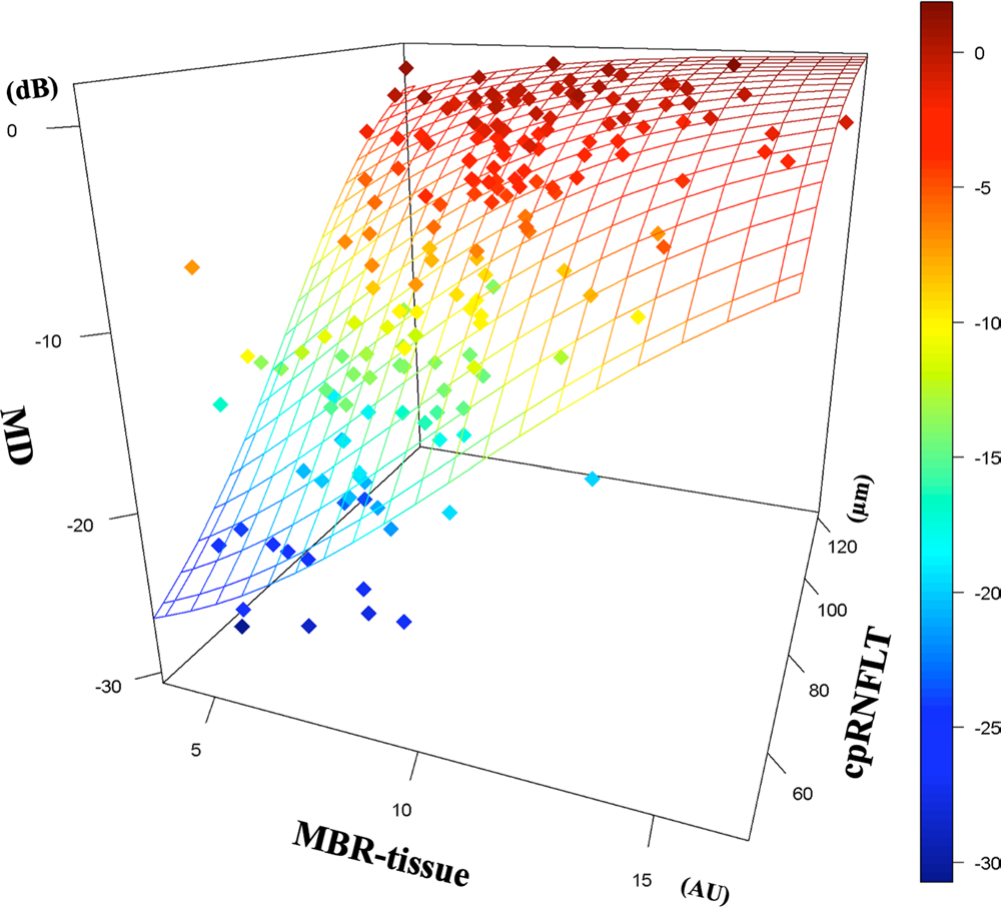
Stepwise multiple logistic regression analyses of the relationships among the mean deviations (MD), the cpRNFLT, and the MBR-tissue. The MBR-tissue and the cpRNFLT were both significant factors markedly correlated with the MD of the visual fields (Odds ratio;1.25(1.03–1.52) and 1.07 (1.02–1.11), respectively). The best model was chosen on the basis of Akaike information criterion (AIC); MD = −30.73 + 32.61/{1 + e^(5.56989-0.06345cpRNFLT −0.22303 MBR-tissue)} (Table 4).

The results of stepwise multiple logistic regression analyses are shown in Fig. 3. The best model was chosen based on the AIC; MD = −30.73 + 32.61/{1 + e^(5.28-0.054cpRNFLT −0.25MBR-tissue)} (Table 4). The curve in Fig. 3 was fit to the MD. The MBR-tissue and the cpRNFLT were both risk factors that were significantly associated with the MD (Odds ratio;1.25 (1.03–1.52) and 1.07 (1.02–1.11), *P* < 0.001 and <0.001 respectively, Table 5).

**Table 4:** Estimate coefficient and standard error (SE) of the explanation variables in the final model following backwards selection of the binary logistic regression.

Factor	Coefficient	SE	Z value	P-value
(Intercept)	−5.56989	1.48184	−3.759	0.000171
cpRNFLT	0.06345	0.02076	3.057	0.002235
MBR-tissue	0.22303	0.09947	2.242	0.02495

**Table 5:** Multivariate logistic regression analysis.

Factor	Odds ratio	95% CI	P-value
MBR-tissue	1.25	1.03–1.52	<0.001
cpRNFLT	1.07	1.02–1.11	<0.001
LogMAR	Not included		
mGCIPLT	Not included		

### Relationships between MD and cpRNFLT and MBR-tissue for each group

**Figure 4:**
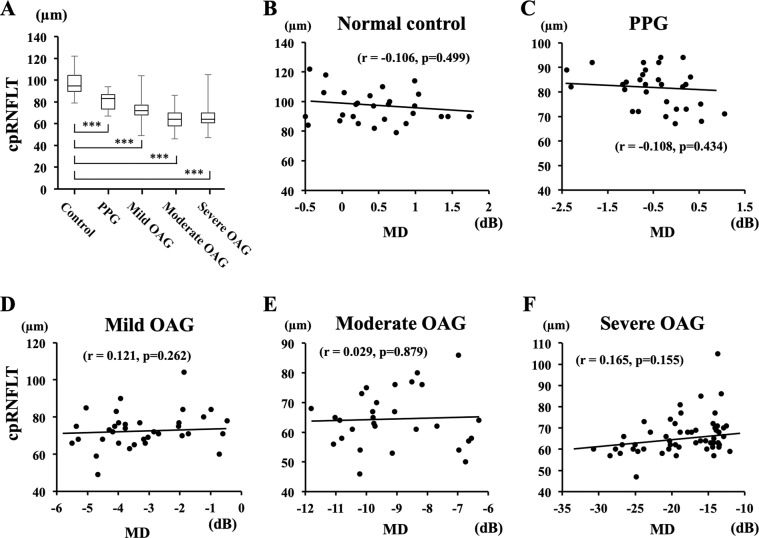
Correlation between the MD and the MBR-tissue for each study group. There were significant correlations between the MD and the MBR-tissue in the mild OAG group (r = 0.444, *P* < 0.001) and the severe OAG group (r = 0.415, *P* < 0.001).

**Figure 5:**
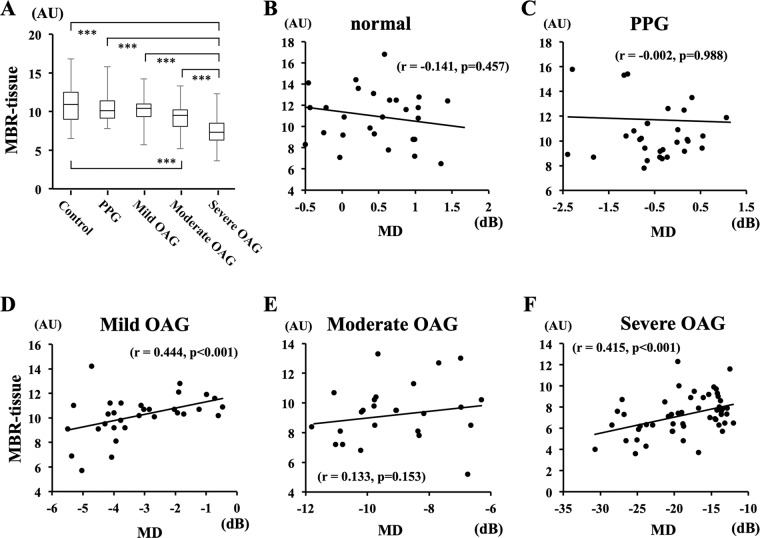
Correlations between the MD and the cpRNFLT for each study group. There was no significant correlation between the MD and the cpRNFLT in any group.

There were significant correlations between the MD and the MBR-tissue in the mild OAG group (r = 0.444, *P* < 0.001) and the severe OAG group (r = 0.415, *P* < 0.001; Fig. 4). However, the correlation between the MD and the cpRNFLT was not significant in any of the groups (Fig. 5).

## Discussion

The results showed that the values of the MD, cpRNFLT, mGCIPLT, and the MBR were reduced in eyes with glaucoma, and the extent of the decrease was dependent on the severity of the glaucoma. Multivariate linear regression analyses showed that the MBR-tissue and cpRNFLT were significant and independent factors that were significantly correlated with the MD. The logistic regression analyses showed that the MD was correlated with the MBR-tissue, the cpRNFLT, and the mGCIPLT. In addition, the analyses showed that the MBR-tissue was the best predictor of the MD based on the AIC. The results of stepwise multiple logistic regression analysis showed that the MBR-tissue and the cpRNFLT were risk factors that were significantly associated with the MD.

Our results showed that the reduction of the cpRNFLT was significantly associated with the severity of the MD, but there were no significant differences in the cpRNFLT in eyes with mild, moderate, and severe OAG. These findings corroborate previous studies that reported that a small additional reduction in the RGC numbers can result in a significant visual field loss at advanced stages of glaucoma^6–8^. Hood *et al*. measured the cpRNFLT in glaucomatous eyes of different glaucoma severities using OCT, and they reported that measurements of the tissue thicknesses were not useful for determining the stage of glaucoma because of the ‘floor effect’, i.e., the RNFLT has a lower limit after which no additional thinning can occur^6–8^.

Mwanza *et al*. used Bayesian methods to estimate the tipping point at which measurable cpRNFL thinning ends, i.e., the cpRNFL reached the floor^14^. The results demonstrated that changes in the SD-OCT values were possible in advanced glaucoma (MD <−12 dB)^15^. Our results showed that the MD was correlated with the cpRNFLT and the mGCIPLT in the logistic regression analysis, but the presence of a floor effect was undetermined. This difference was probably due to differences in the calculations and number of subjects in the different reports^6–8,14^. However, although a relatively larger number of estimated RGCs should be lost in earlier glaucoma, a small number of RGCs should still be lost even in advanced glaucoma^2,3^. In addition, the examinations of the structural changes of the cpRNFLT reflect the loss of retinal ganglion cells, but the thickness probably does not solely directly reflect the residual nerve fibers on the optic disc. Although the cpRNFLT should be a good factor to use to detect the earlier stages of glaucoma, e.g., PPG, it is most likely not a clinically useful biomarker for advanced glaucoma.

Our results showed that the MBR-tissue was the best factor to predict the MD using logistic regression analysis, and multiple regression analysis showed that the MBR-tissue was the factor most significantly associated with the MD. In addition, the MBR-tissue was significantly correlated with the MD even in eyes with advanced glaucoma. These results suggest that the blood flow parameters may be useful biomarkers for the classification of the severity of the glaucoma even at the advanced stages of glaucoma.

There have been many studies that reported that the ocular blood flow is decreased in eyes with glaucoma^16–19^. In addition, many studies have suggested that the ONH blood flow plays a critical role in the pathophysiology of glaucoma because low ocular blood flow can lead to RGC damage by stimulating the production of cytotoxic substances by the astrocytes and microglia^20^. In addition, the impairment of the ONH blood flow affects the mitochondria in the RGCs directly resulting in less ATP available to the cells thus attenuating their axonal transport^21^.

Yokoyama *et al*. reported that the average MBR-all was significantly lower in 60 glaucomatous eyes than in the control group. This validates the rationale of using LSFG in glaucoma patients^22^. More recently, there have been reports that the MBR-tissue is useful for identifying earlier stages of glaucoma in humans^13,23^ as well as in an experimental animal model of glaucoma^24^. The MBR-tissue corresponds mainly to the capillary network on the ONH, and thus, the MBR-tissue impairment should be a good candidate for predicting the severity of the glaucoma.

The multivariate regression analysis showed that the reduction of blood flow and structural alterations were both significant and independent predictors of the MD in the glaucoma patients. These findings suggest that structural evaluations alone can only provide partial information on the severity of the disease. The blood flow and structural evaluations have allowed us to evaluate the severity of the glaucoma more accurately.

The results also showed that the MBR-tissue was significantly correlated with the cpRNFLT indicating that the blood flow was closely related to the structural changes and subsequent visual field defects. There are several possibilities to explain the significant association between the blood flow reduction and structural changes of the optic disc and visual field defects. First, in eyes with clinically detectable glaucoma, the ONH has already undergone significant structural changes with the loss of nearly 50% of the retinal nerve fibers^25^. It is possible that the loss of neural tissue is related to the reduction in blood flow by a decrease in the metabolic need and visual field defect^11^. Second, the structural alterations and visual field defect may be the consequence of the ischemic damages due to the perfusion deficits. Third, the visual field loss may arise from the reduced blood flow that is independent of the structural loss. Additional experiments are needed to determine which of these changes has occurred.

There are limitations in this study. First, the study used a non-interventional case-control design. Second, the glaucoma and normal groups were age-matched, and there were no significant differences between the groups for a history of hypertension, and ocular perfusion pressure. However, there was a significant difference in the choroidal MBR, the use of anti-thrombotic and glaucoma medications, and the ratio of intracranial disease and diabetes mellitus. Therefore, there may be confounding elements that remain unidentified and the potential impacts of both ocular and systemic medications warrants further investigation. Third, this study assessed the blood flow on the ONH and not the retinal flow. The loss of the RNFL is more localized in mild to moderate glaucoma, and there may be significant correlations between localized RNFL loss and localized retinal flow. Fourth, our results showed that there were significant differences in the sex and lens status distribution. It has been reported that the MBR on the ONH in women is faster than that in men^26,27^. The ratio of men in the severe OAG group was higher than the other groups in our study, and it may have affected the results. However, the MBR-tissue in the severe OAG group was more than 20% lower than the other groups, which was much larger than the reported difference between men and women^26,27^. Although eyes with severe cataract were excluded from the study, the presence of these eyes may have affected the results of the MBR or the MD. In our study, the relatively small sample size for each group may limit the ability to detect small differences between the groups. Further prospective investigations using groups with a consistent background and a larger number of subjects are needed to determine the relationship between the blood flow reduction and the structural changes.

In conclusion, the MBR-tissue is the best factor for identifying the alterations of the MD of the visual fields. In addition, the blood flow reduction and structural alterations were both significant and independent predictors of the visual field loss in glaucomatous eyes. These results indicate that the blood flow measurements would potentially be as important as structural changes in the diagnosis and prognosis of glaucoma.

## Patients and Methods

### Ethics statement

This study was conducted in adherence with the tenets of the Declaration of Helsinki and an informed consent had been obtained from all of the patients. This was a retrospective, observational comparative, single-center study, and the procedures were approved by the Institutional Review Board and the Ethics Committee of the Nagoya University Graduate School of Medicine.

### Subjects

We reviewed the medical records of all patients who were diagnosed solely with open-angle glaucoma by glaucoma specialists at the Nagoya University Hospital from November 2013 to December 2017. When both eyes met the inclusion criteria, one eye was randomly selected for the evaluations and analyses. The normal eyes were the fellow eyes of age-matched patients who visited to our hospital to treat the other eye for rhegmatogenous retinal detachment or epiretinal membrane. The inclusion criteria for the normal eyes were: (1) normal findings in slit-lamp and ophthalmoscopic examinations; (2) best-corrected visual acuity (BCVA) better than 20/25; (3) IOP ≤21 mmHg; and (4) visual field within the normal limits of the Anderson–Patella classification. The inclusion criteria for the PPG eyes were: (1) normal open angle by gonioscopic examination; (2) BCVA better than 20/25; (3) IOP less than 22 mmHg; (4) glaucomatous changes of the optic disc including a thinning of the neuroretinal rim, notching and cupping; (5) visual fields within the normal limits of a glaucomatous hemifield test, i.e., with pattern standard deviation (PSD) greater than 5%, confirmed in at least two examinations; and (6) an abnormal thinning of the retinal nerve fiber layer in at least one clockwise OCT scan sector, at 6, 7, 8, 10, 11, or 12 o’clock, confirmed in at least three examinations. The inclusion criteria for the OAG group were; (1) presence of glaucomatous optic disc changes determined by biomicroscopy and visual field defects and abnormal cpRNFL thinning as determined by Cirrus OCT with embedded software, (2) IOP ≤21 mmHg in at least three examinations, (3) open angle by gonioscopy, (4) age >20 years, (5) refractive error between +3.00 to −8.00 diopters, and (6) best-corrected visual acuity (BCVA) ≥20/30.

### Exclusion criteria

The exclusion criterion was a history of ocular or systemic disease affecting the ONH, and an elevated IOP. In addition, eyes with a BCVA <20/25, IOP >21 mm Hg on the test day, severe cataract, and high myopia, i.e., axial length (AL) longer than 27 mm were also excluded.

When both eyes met the inclusion and exclusion criteria, one eye was randomly selected for the evaluations and analyses.

### Measurements of clinical parameters

All subjects underwent ophthalmologic and general examinations that included; slit-lamp and ophthalmoscopic examinations, gonioscopy, IOP measurements, systemic blood pressure measurements, perimetry, OCT examinations, and ONH blood flow measurements. The decimal BCVA was converted to the logarithm of the minimum angle of resolution (logMAR) units for the statistical analyses. The axial lengths were measured by partial optical coherence interferometry (IOLMaster; Carl Zeiss Meditec, La Jolla, CA), and the IOP was measured with a handheld tonometer (Icare; TiolatOy, Helsinki, Finland). The SBP and the DBP were measured with an automatic sphygmomanometer (CH-483C; Citizen, Tokyo, Japan). The MAP and MOPP were calculated as follows: MAP = DBP + 1/3(SBP-DBP), and MOPP = 2/3MAP  −  IOP.

### Perimetry

The visual fields were determined with the Humphrey Field Analyzer II (HFA; Carl Zeiss Meditec AG, Jena, Germany) using the Swedish interactive threshold algorithm (SITA) standard central 30-2 program. The MD was recorded as an objective measure of the visual field, and only reliable MD values were used which excluded examinations with 20% fixation errors and >33% false- positives or false-negatives^22,28^. The eyes in the OAG group were placed into three groups according to the degree of visual field impairment: mild, MD >−6 dB; moderate, MD between −6 and −12 dB; and severe, MD <−12 dB.

### Measurement of circumpapillary retinal nerve fiber layer thickness (cpRNFLT) and ganglion cell layer plus inner plexiform layer thickness (mGCIPLT)

The cpRNFLT and mGCIPLT were measured using the manufacturer’s software with a SD-OCT system (Cirrus, Carl Zeiss AG, German). Poor quality images caused by eye movements, blink artifacts, poor centration, and signal strength <7 were excluded from the analyses.

### Laser speckle flowgraphy (LSFG)

The LSFG-NAVI (Softcare Co., Ltd.) instrument was used to determine the ONH blood flow. The principles of LSFG have been described in detail^29–32^. Briefly, this instrument consists of a fundus camera equipped with an 830 nm diode laser as the light source and a standard charge-coupled device sensor (750 width × 360 height pixels) as the detector. After switching on the laser, a speckle pattern appears due to the interference caused by the light scattered by the movements of the erythrocytes. The MBR is a measure of the relative blood flow velocity, and it is determined by examining the pattern of the speckle contrast produced by the erythrocytes in the ocular blood vessels. The MBR images are acquired at a rate of 30 frames/s over a 4-s period. The same site can be measured by using the auto-tracking system. To evaluate the circulation on the ONH, a circular marker is set surrounding the ONH (MBR-all). The “vessel extraction” function of the software then identifies the vessel and tissue areas on the ONH so that the MBR of each area will be assessed separately. The MBR of the vessel, the MBR-vessel, and tissue, MBR-tissue, on the ONH were determined. The LSFG was measured two times at each time point in all eyes. The average of the MBRs was calculated and used for the statistical analyses.

Subjects were instructed to abstain from alcohol and stimulating beverages containing caffeine (e.g. tea, coffee) 12 hours before the LSFG measurements, as these are known to potentially influence the results^28^. The measurements were performed in a quiet, dark room with the subject in a sitting position from 9 AM to 1 PM to preclude any effect of diurnal variations^33^.

### Statistical analyses

Chi-square tests were used to compare the categorical data, and Kruskal-Wallis tests were used to compare continuous variables among the groups. Linear regression analysis was used to determine the correlation between MD and blood flow parameters, i.e., the MBR-tissue, the MBR-vessel, and the MBR-all, or the structure parameters. i.e., the cpRNFLT and mGCIPT. Stepwise multivariate linear regression analyses were used to determine the correlations between the MD and the MBR-tissue, cpRNFLT, BCVA, mGCIPLT, age, MOPP, AL, and IOP. Logistic regression analyses were used to determine the correlation between the function and structure, in particular the cpRNFLT, mGCIPT, and MBR-tissue. Stepwise multiple logistic regression analysis was used to select the best model on the basis of the AIC. The curves in Figs. 2 and 3 are the best fit curve determine by regression analyses. To be specific, the normalized mean deviation of visual field,

$${\rm{MD}}={\rm{a1}}+{\rm{a2}}/(1+{{\rm{e}}}^{(-{\rm{f}})}),$$

where f = b0 + b1*cpRNFLT + b2*mGCIPLT + b3*MBR-tissure; b1, b2, and b3 are the coefficient of each parameters attributable to MD; a1, a2, and b0 are the intercepts. A *P* < 0.05 was considered statistically significant. All statistical analyzes were performed with R version 3.5.0.
